# Therapeutic Signature of Stem Cell Derivative Exosomes in Oral Cancer: A Scoping Review

**DOI:** 10.7759/cureus.39957

**Published:** 2023-06-04

**Authors:** Aanuja Singha, Magesh K T, Ramya Mahalingam, Sathya Kumar M, Aravindhan R, Sivachandran A

**Affiliations:** 1 Oral Pathology and Microbiology, SRM Institute of Science and Technology, SRM Kattankulathur Dental College and Hospital, Chennai, IND

**Keywords:** biogenesis of exosomes, cancer drug delivery and epithelial mesenchymal transition, stem cell, exosomes, oral cancer

## Abstract

Oral cancer poses a serious health challenge to the nations worldwide. India, among all the nations reported, has the largest number of oral cancer cases, which accounts for one-third of the entire population of oral cancer globally. As oral cancer is well-known for delayed diagnosis until an advanced stage, poor outcomes, and a lack of specific biomarkers for the disease and high-budget therapeutic alternatives.

Stem cell derivative exosomes gained significant attention as therapeutic agents and diagnostic biomarkers in cancer biology. It's a type of extracellular vesicle, which are lipid-bilayer-enclosed vesicles of endosomal origin. They are nanosized membrane vesicles that are capable of self-renewal, unlimited proliferation, and multi-directional differential potential. Thus, they act salient in the occurrence and development of tumors. Exosomal micro-RNAs (miRNAs) are functionally related to the advancement of cancer, metastasis, and the aggressive nature of tumors with high recurrence rates.

It has also been highlighted that exosomes have the potential to serve as diagnostic markers. A quick, easy, high-clarity, and confined rehabilitation method is the basic specification for large-scale usage of exosomes. The constitution of the composite transporters of exosomes is easily available by sampling biological fluids (liquid biopsies) from samples such as saliva. A liquid biopsy based on exosomes focuses on their probable usage in cancer patients' diagnosis and the determination of the outcome or course of the disease.

This review explores the therapeutic prospect of stem cell-derived exosomes as intending to offer new ideas for clinical management and institute a new era of therapeutic agents for oral cancer.

## Introduction and background

Cancer is a wide terminology that depicts a group of conditions that out-turn from gene alterations, resulting in cellular changes that are attributed to mutations that bring about uncontrolled growth and cell division. Among cancer, oral cancer is one of the most prevailing types of cancer worldwide and globally ranks sixth of all types [1].

Oral cancer refers to a subpopulation of head and neck cancer that consists of cancers of the lip mucosal surface, the floor of the mouth, tongue, buccal mucosa, gingival mucosa, and hard or soft palate.

Oral cancer is otherwise known as carcinomas as their incidence is more than 90% and histopathology represents squamous differentiation from the mucosal epithelium, thus named oral squamous cell carcinoma (OSCC). This cancer is a genetic and epigenomic disease, distinguished by multifactorial etiology with various risk factors, which include smoking tobacco, consumption of alcohol, presence of potential malignant or pre-malignant oral lesions, human papillomavirus (HPV), malnutrition, radiation, and hereditary tendency. Among all the probable risk factors, tobacco and alcohol consumption are dominant contributors to causing oral cancer. Heterogenicity in oral carcinogenesis takes part in a significant role in the tumor microenvironment regarding tumor and non-tumor cells, making tumors resistant to conventional therapies [2].

Despite the extensive progress in conventional therapeutic approaches, many adverse effects accompanied them, e.g. surgical resection of the tumor, chemotherapy, or radiotherapy alone or in combination. Surgical resection is the treatment of choice, but results in irreversible deformity, changed self-perception, significant functional limitations, and overall patient well-being. Even though the treatment outcomes and therapeutic advances improved, the prognosis has not been significantly improved with a five-year survival rate of upto 50%-60% as a consequence of delayed disease detection until advanced stages, with the likelihood of localized recurrence and distant metastasis [3].

Thus, to enhance individual health outcomes and survival, it is crucial to develop novel therapeutic approaches, while early tumor detection remains a primary concern and a major issue to be resolved.

Exosomes are nano-sized, extracellular, membrane-enclosed vesicles, averaging 100 nm in diameter. These vesicles release from the majority of cell types, including stem cells, tumor cells, and biofluids such as saliva, amniotic fluid, blood, and cerebrospinal fluid [4-7]

Recently, they have gained a lot of attention as an innovative drug delivery nanoplatform. The known function of exosomes as transporters that carry cargo, such as proteins, lipids, DNA, mRNA, non-coding RNA, and miRNA, acts as a significant tool for intercellular communication and signal transduction. This influences the tumor microenvironment, mediating tumorigenesis, immune resistance, metastasis, angiogenesis, and resistance to therapy, implicating the clinical potentiality of exosomes as prognostic and diagnostic biomarkers and therapeutic agents [8,9].

This presents the urgent need for improvement in the therapeutic regimen of current approaches, which is to develop an effective targeted drug delivery system to overcome the drawbacks of conventional treatment strategies.

An innovative approach of stem cell-derived exosomes as a targeted drug delivery complex provides the excellent potential to improve drug bio-absorbability and bio-distribution at the site of the tumor. Therefore, implementing novel strategies can be useful in promoting anticancer therapy.

## Review

We collected data from 27 articles published mainly in PubMed and Google Scholar using keywords like oral cancer, exosomes, and stem cell-derived cancer drug delivery.

**Figure 1:**
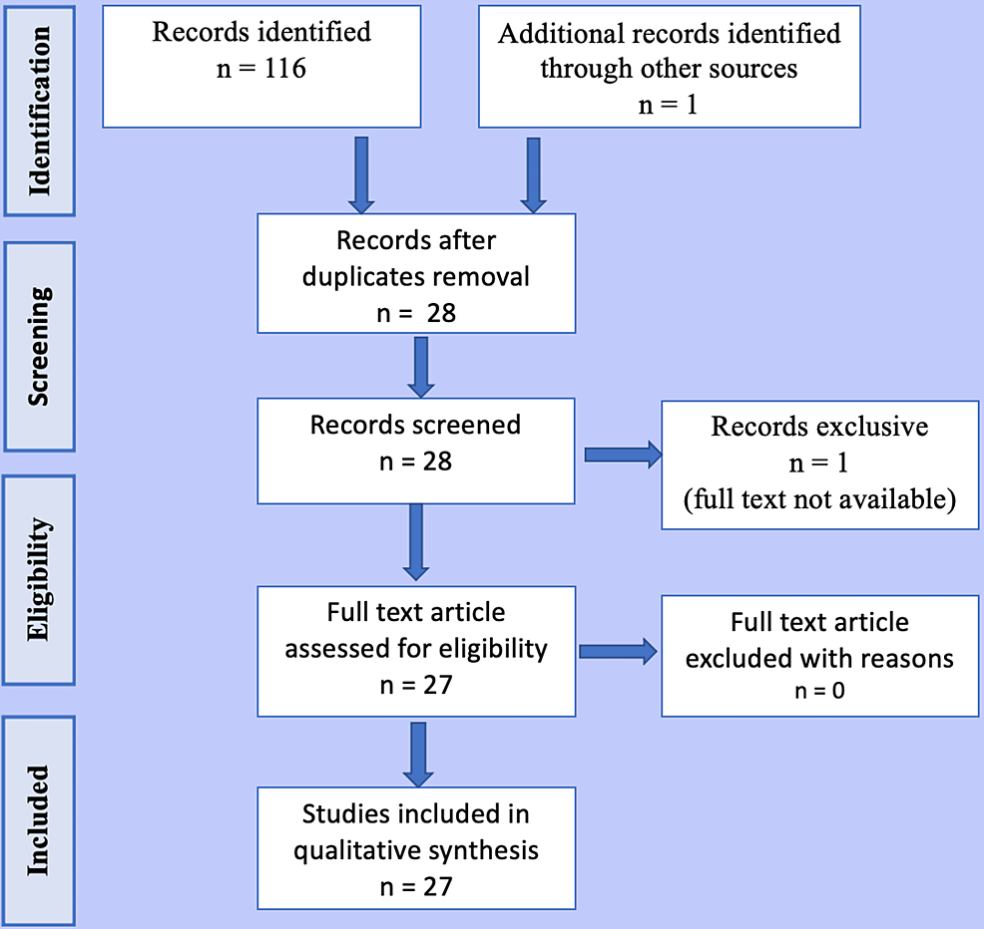
The Preferred Reporting Item for Systematic Reviews and Meta-Analyses (PRISMA) protocol

The Preferred Reporting Item for Systematic Reviews and Meta-Analyses (PRISMA) protocol was adopted (Figure 1). 

Biogenesis of exosomes

The intraluminal vesicles (ILVs) of multivesicular bodies (MVBs) that occur along the endocytic pathway give rise to exosomes that then fuse with the cell surface of late endosomes. Vesicle formation takes place inside the lumen (ILVs) following the inward budding from the limiting membrane of late endosomes, which derives a particular type of late endosome known as MVB through the accumulation of hundreds of ILVs [10,11].

The fate of MVBs in the biogenesis of exosomes depends on different sorting processes, which either lead to autophagosomes or exosome production following fusing with the cell membrane in the extracellular environment [12].

The incorporation of exosome-specific markers or endogenous molecules aids in exosome identification in the course of biogenesis such as membrane proteins tetraspanins (CD9, CD63, CD81, and CD82), tumor necrosis factor receptor 1 (TNFR1), apoptosis-linked gene 2-interacting protein X (ALIX), TSG101, docking and membrane fusion (RABs, ARF), phospholipids, ceramides, sphingomyelinases, and SNARE (soluble N-ethylmaleimide-sensitive factor (NSF) attachment protein receptor) and heat shock proteins (Hsp90, Hsp70, and Hsp60) [13].

Exosomes are also shown to be affected by external agents such as viral or non-viral and microbial infections, Leishmania, and Trypanosoma cruzi [14].

There are at least two different routes that mediate the process of MVB biogenesis, which involves the sorting of various molecules into ILVs [15,16].

The endosomal sorting complexes required for transport (ESCRT)-dependent mechanism consist of around 33 proteins, which are finally grouped into four proteins complexes as ESCRT-0, -I, -II, and -III in addition to various accessory proteins such as VPS4, VTA1, tetraspanin-enriched domains, sphingomyelinases, and ALIX [11]. ESCRT-0 enables cargo to cluster transfer and enlists other ESCRT protein complexes, such as ESCRT-I, ESCRT-II, and ESCRT-III, to the endosomal membrane [14]. In order to facilitate mono-ubiquitylated cargo proteins, it is required to involve a constituent of ESCRT-0, hepatocyte growth factor-regulated tyrosine kinase substrate (HRS) with signal transducing adapter molecule 1 (STAM1), clathrin, and Eps15 to form a complex. Afterward, HRS interacts with TSG101 to recruit the ESCRT-I complex. ESCRT-I and ESCRT-II incite a high affinity of endosomal inward budding around ubiquitinated clusters of proteins. ESCRT-III in the final stage facilitates intraluminal vesicle formation through binding with ESCRT-II [15]. The de-ubiquitylating enzymes (DUBs) cause the cargoes to become de-ubiquitinated if the ILVs do not undergo degradation by the lysosomes. In the course of dissociation and recycling of the ESCRT-dependent pathway, the participation of auxiliary proteins, such as VPS4 ATPase, plays a crucial role [10,17-19].

Accumulating several studies, despite the depletion of ESCRT protein complexes, unlike in the ESCRT-dependent pathway, ILV formation in MVB and exosome biogenesis occurs suggesting an alternating pathway as an ESCRT-independent mechanism. This mechanism involves proteins, lipids, and tetraspanins [18].

The lipids involved in the biogenesis of exosomes are targeted by specific lipid-modifying enzymes. The plasma membrane of endosomes contains lipid-enriched parts such as sphingomyelin and ceramide. Ceramide induces the inward budding of the plasma membrane of MV to form ILVs. Neutral sphingomyelinase 2 (nSMase2) enzyme inhibition disintegrates sphingomyelin to generate ceramide, resulting in the reduced release of proteolipid protein (PLP) bearing exosomes secreted by oligodendroglial cells. Thus, nSMase2 promotes inward budding and the formation of ILV and MVB production, which is controlled by Rab GTPases either destined for degradative or secretory pathways.

Another protein also reported to take part in exosome biogenesis is known as the small integral membrane protein of the lysosome/late endosome (SIMPLE) called lipopolysaccharide-induced TNF factor or LITAF [12].

The ESCRT-independent pathway was discovered to be helpful in MVB and exosome genesis, exosome content modification, and developing new therapeutic strategies.

Therefore, both pathways might not be completely separated but rather work synergistically, and various exosome subsets could depend on different mechanisms [20].

Interlinking of exosomes and oral cancer

Exosomes, a type of extracellular vesicle (EV) considered a waste cell entity, are formed by cellular damage. Recent studies have shown the involvement of exosomes in biological functions in cells, pathological conditions, especially cancer, and mediated intercellular communications. Exosomes, therefore, serve a dual-edged role in the immunity to oral cancer [4]. The lipid bilayer exosomes contain various cargos including mRNAs, miRNAs, and noncoding RNAs (ncRNAs) such as circular RNAs, transfer RNAs, ribosomal RNAs, and nucleic acids. These exosomal components regulate uncontrolled cell growth, angiogenesis, metastasis, chemosensitivity, and immune evasion. Currently, exosomes depict the hallmark for oral cancer diagnoses and prognosis biomarkers, minimally invasive liquid biopsies, and potential therapeutic drug therapies [1,5].

The most prevalent head and neck cancer is oral squamous cell carcinoma (OSCC) with a high metastatic invasion rate. In spite of the fact that treatment outcome has been improved for OSCC, the less therapy efficacy and poor prognosis result in infeasible curative resections due to late diagnosis until an advanced stage. In view of this, early diagnosis, improvement in prevention, and the treatment efficacy of oral cancer are key goals in epidemiological cancer control and management.

Exosomes and angiogenesis

**Figure 2:**
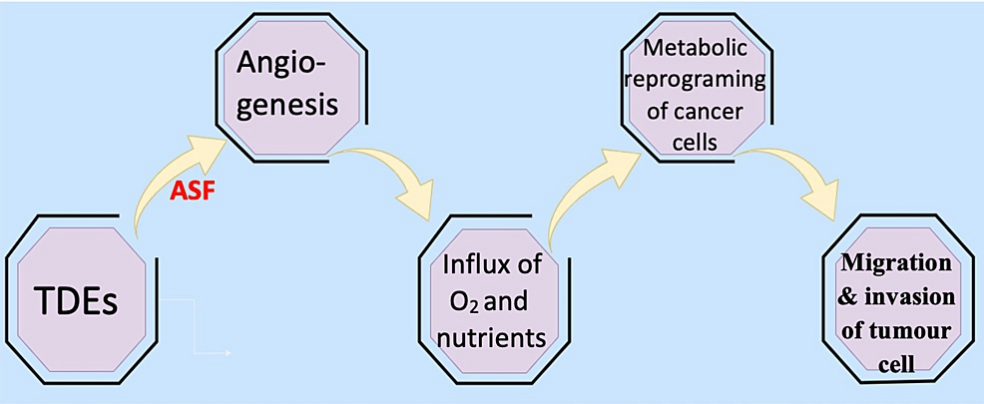
Steps involved in angiogenesis associated with TDEs TDE: tumor cell-derived exosomes

Tumor cell-derived exosomes (TDEs) have features such as host immune modulation and contact with tumor microenvironment (TME) networking. Recent studies have shown that TDEs can support angiogenesis, an important component to initiate vascularisation that helps in tumor progression. Angiogenesis is a crucial multi-phase process for tumors to develop and metastasize by developing new vasculature (Figure 2).

The angiogenic-stimulating factors (ASF) by TDEs are vascular endothelial growth factor (VEGF), fibroblast growth factor (FGF), platelet-derived growth factor (PDGF), transforming growth factor β (TGF-β), tumor necrosis factor α (TNF-α) and interleukin-8 (IL-8) [21]. Among all the factors involved, the VEGF receptor signaling pathway is the most appropriate angiogenic target.

Exosome-mediated cancer immunosuppression

Exosomes can also possess an immunosuppressive effect by preventing proliferation and causing T-cells apoptosis by sending death signals via the FAS/FASL pathway [4].

**Figure 3:**
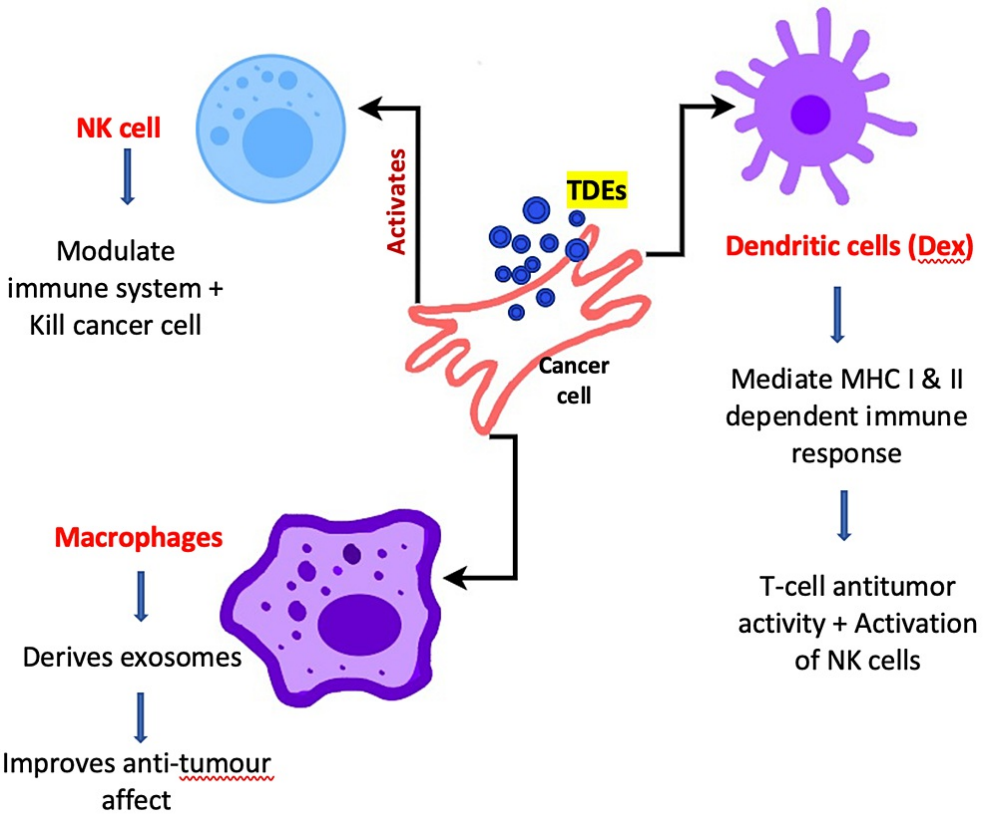
Effect of TDEs on immune cells TDEs: tumor cell-derived exosomes

Immune cells, such as dendritic cells, natural killer (NK) cells, and macrophages, produce exosomes that mediate antitumor activity and the proliferation and activation of NK cells, which in turn modulates the immune system and destroy the invading cancer cells (Figure 3) [22]. 

MSC exosomes derived from the oral mucosa possess superior immunomodulation and may inhibit tumor growth by increasing inflammatory infiltration, reducing angiogenesis, and decreasing signaling pathways linked to tumors.

Exosomes and epithelial-to-mesenchymal transition (EMT)

For the migration of tumor cells to distant sites, a new metastatic niche is needed. For metastasis to happen, a process must facilitate the transmission of tumor epithelial cells; this is called the epithelial-to-mesenchymal transition (EMT) process. The essential elements of EMT accompanied by TDEs are TGF-β, HIF1α, β-catenin, IL-6, caveolin-1, or vimentin and nucleic acids like EMT-inducer miRNAs. Tumor cells lose their epithelial characteristics and transform to mesenchymal features by losing E-cadherin, cell-cell adhesion, and apicobasal polarity while obtaining N-cadherin, twist, snail, and vimentin. Consequently, cells that have undergone EMT develop aggressive features and acquire characteristics similar to stem cells [4].

Exosomes and extracellular matrix (ECM)

**Figure 4:**
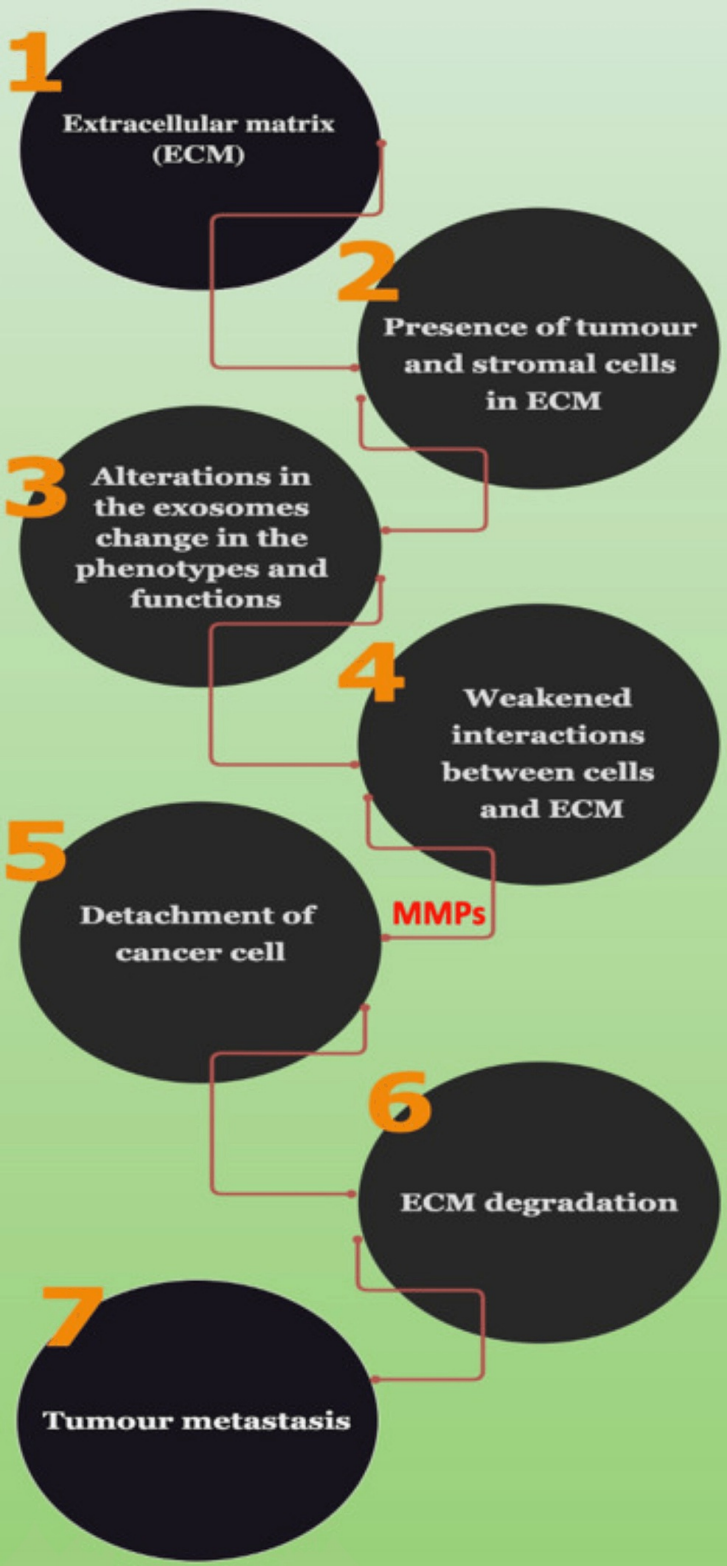
Steps involved in the degradation of the ECM by the TDEs EMT: epithelial-to-mesenchymal transition; ECM: extracellular matrix

The ECM is linked to the changes in the phenotype and function of tumor cells and stromal cells. It is believed that the exosomes of cancer cells have a degrading effect on the ECM, which, in turn, facilitates the metastasis of the cancer cells (Figure 4).

Therefore, the correlation between exosomes and the tumor microenvironment by mediating ECM remodeling and eventually leading to tumor metastasis is shown [4].

TDEs as promoters of organ-specific metastasis

In 1889, Paget put forward the ‘seed and soil’ hypothesis that depicts that tumor cells (seeds) tend to metastasize to specific organs (soils), which suggests that tumor metastasis of the target organ is not an arbitrary option [23]. For cancer to metastasize, the contribution of a pre-metastatic niche must exist. Certain integrin patterns, such as α6β1, α6β4, αvβ5, and αvβ3, exhibited by exosomes are linked to specific cell types in target organs and help predict potential pre-metastatic niches at organotrophic locales. Additionally, adhesion molecules and other elements present on the exosomal surface may facilitate exosome cell fusion and selective adherence to sites of potential metastasis, which would promote organotropism [4].

This depicts that the release of exosomes from tumors contributes to immune repression and accelerates tumor spread and growth. Furthermore, in the tumor microenvironment, immunoinhibitory (protumor) and immunostimulatory (antitumor) signals allow TDEs to interact with immune cells.

Exosomes as a cancer diagnostic biomarker

Several studies revealed miRNAs and ncRNAs found in exosomes of tumors are involved in cancer initiation and progression, which may serve as diagnostic tools.

Exosomal miR‐486 and miR‐10b‐5p have been detected in patients with head and neck squamous cell carcinoma (HNSCC) at increased levels in their saliva. There are findings that show a significant increase in salivary nitrosamines and heavily oxidized salivary DNA and proteins while, on the other hand, a decrease in salivary antioxidants. These could explain the link between oxidative damage to the DNA and proteins by free radicals and antioxidants present in saliva with OSCC. Moreover, studies have shown a significant rise in salivary miRNA-21 and miRNA-184 in OSCC and PMD in contrast to diseased and healthy controls. This demonstrates the significance of non‐invasive salivary biomarkers of HNSCC from exosomal miRNAs.

The liquid biopsies can have the clinical potential application of circulating tumor DNAs (ctDNA), ctRNA, circulating tumor cells (CTCs), long non-coding RNAs, and exosomal miRNAs in HNC.

It has been proved that exosomes derived from MSCs and exosomal miR-8485 are involved in oral, potentially malignant disorders (OPMD), which include oral leukoplakia, erythroplakia, oral submucous fibrosis, and carcinogenesis, hence by intruding in the production of exosomes derived from MSCs could lead to an innovative approach that intercepts or reduces the malignant transformation [24].

Sources of stem cell-derived exosomes in biomedical applications

Stem cells derived from oral tissue currently received more recognition because they are a rich source of cells and can be obtained through non-invasive procedures (Table 1) [25].

**Table 1:** Sources of stem cell-derived exosomes

Source	Application
Dental pulp stem cells (DPSCs)-derived exosomes	Increased mineralization and regeneration of tissue, immunomodulation, and angiogenesis
Stem cells from human exfoliated deciduous teeth (SHED)-derived exosomes	Odontogenic differentiation, bone regeneration, anti-inflammation (suppress carrageenan-induced acute inflammation)
Periodontal ligament stem cells (PDLSCs)-derived exosomes	Immunomodulation, osteogenic differentiation, angiogenesis
Stem cells from apical papilla (SCAPs)-derived exosomes	Promotion of dentinogenesis
Gingival mesenchymal stem cells (GMSCs)-derived exosomes	Osteogenic differentiation, anti-inflammation, bone regeneration, and taste bud regeneration

Stem cell-derived exosomes in oral cancer drug delivery

The capability of exosomes as an ideal drug delivery carrier has been considered effective for various diseases, including cancer, on account of minimum cytotoxicity, good biodistribution, and maximization of the bioavailability of the drug. They also do not induce immune rejection or other complications caused by relatively non-immunogenic properties and moreover possess an intrinsic ability to cross biological barriers, chiefly the blood-brain barrier (BBB).

Various cell types have been analyzed, including immature DCs, macrophages, and cancer cells, for the isolation of drug carrier exosomes. Exosomes helped increase the bioactivity and achieve targeted drug delivery by incorporating therapeutic agents, proteins, short interfering RNAs (siRNAs), and targeted drugs via electroporation, sonication, parental cell modification, or direct incubation [5].

After the integration of therapeutic agents with exosomes, they enter the targeted recipient cell where the anticipated cargo is released. The components of cargo involved in drug delivery are responsible for heterogenicity in biological processes such as gene expression, immune complex response, and signal transduction. A similar mechanism is assimilated to combat cancer, in which exosomes where loaded with therapeutic drugs, antibodies, or RNA for the manipulation of gene expression of the disease. Moreover, TDEs promote therapy resistance by improving DNA repair or distributing transporters to treatment-sensitive cells. Exosomes also aid in tumor microenvironment-associated treatment assistance as moderators of mesenchymal stem cells and EMT [26].

The occurrence of oral SCC in decreased oxygen microenvironment advances the generation of miR-21-rich exosomes by hypoxia-activated factors, HIF-1a and HIF-2a, and these miR-21-rich exosomes meditate relocation and breaching behaviors. Then, miR-21 expression reinstitutes in HIF-1a and HIF-2a decreased exosomes, which prevents further oral SCC relocation and breaching.

The therapeutic technology has been significantly escalated by the advancement of RNA interference in biomedical applications. The targeted mRNA in the disease is selectively suppressed by transferring siRNA to recipient cells, showing a great contribution to treatment outcomes. On the other hand, it has been found that naked siRNA quickly degrades by nucleases present in blood circulation, which, due to their negatively charged surfaces, may not enter target cell membranes. Thus, siRNAs encapsulation in exosomes gives promising novel strategies to overcome most of these delivery hurdles [27].

Future perspectives

The realm of biomedical science in exosomes derived from oral tissue stem cells is impending with interest and importance. The recent evidence in this field encourages innovative cell-free therapy as a promising treatment for various diseases and regenerative medicine. Further, thorough research is inevitable to know the molecular biology involved between exosomes from different cells and from the same cell type in the necessary environment.

In the last few years, interest has profoundly expanded in regard to the mechanisms and roles of exosomes in OSCC and OPMD. While the understanding of exosomes as an integral part of the intercellular complex increases, the isolation of exosomes from the oral cavity could potentially come up with unique, easy, and direct access to the tumor microenvironment [3].

With advanced studies on the role and characteristics of exosomes in oral diseases, such as OSCC, it is important to understand exosomes influencing angiogenesis, metastasis, tumor phenotype, immune modulation, and drug refusal. There are still several questions that need to be explored and analyzed.

Exosomes unfold a new chapter in drug load delivery systems with their low immunogenicity and excellent biocompatibility. The versatility of exosomes' biological functions could be promising tools in diagnosis and prognosis, which help in further studies to emerge as a next-generation nanoplatform for therapeutic purposes [28].

## Conclusions

Growing studies and research on exosome-derived stem cells led to the belief of exosomes as significant diagnostic and therapeutic regulators of several oral diseases. The unique advantages that exosomes possess over derivative stem cells (DSCs) are high specificity, lower immunogenicity, highly biocompatible, easily procured, low side effects, high drug load capacity, and nanoscale size, which leads toward regenerative medicine. In view of this, the biology of exosomes, especially in oral diseases such as cancer, is coming to light and the number of studies addressing their value has increased substantially. Moreover, exosomes are in view for the regulation of many biological processes such as cell-to-cell communication, anti-inflammation, angiogenesis, osteogenesis, immune system modulation, and promotion of tumor cell apoptosis. Thus, the accurate comprehensive pathways involved in biogenesis, isolation methods, and sources of exosomes are important for diagnostic, prognostic, and therapeutic reasons. Knowledge regarding the heterogenicity of EVs, their cargo, and endogenous transport functions will evolve toward therapeutic value as the need for precise characterization of exosomes grows simultaneously. Therefore, exosomes can serve as an ideal diagnostic and prognostic biomarker for HNSCC and other diseases due to their unique secretion pattern. The potentiality of exosomes as drug delivery systems is another substantial contribution to be considered. Exosomes fusing with the cell membrane can be of high reliability, therefore allowing the delivery of peptide drugs or non-coding RNAs to tumor cells through exosomes. There is still undiscovered scope in the expanse of exosomes in biomedical applications. However, on the contrary, the exosome mechanism involved in the occurrence, metastasis, proliferation, immunosuppression, and chemotherapy resistance of HNSCC should not be unacknowledged. This opens a window of hope for allowing multicompetent diagnostic disease detection and monitoring. To sum up, this review outlines that exosomes have gained and come into focus as natural nanoparticles with properties ideal for diagnostic, prognostic, and therapeutic biomarkers in oral diseases.
